# Harnessing Multi-Anchoring Effects for the Fabrication and Specific Recognition of Surface-Oriented Imprinted Nanospheres for Cytochrome C

**DOI:** 10.3390/polym18101261

**Published:** 2026-05-21

**Authors:** Nan Zhang, Yang Qiao, Kaishan Yu, Jinrong Zhang, Pengfei Cui, Chengzhao Yang, Minglun Li

**Affiliations:** 1 College of Chemistry and Chemical Engineering, Xi’an University of Science and Technology, Xi’an 710054, China; lindajun2022@163.com (Y.Q.); 18709496409@163.com (K.Y.); zhangjinrong0503@163.com (J.Z.); c1848018709@163.com (P.C.); yyyylolol@163.com (C.Y.); 2 State Key Laboratory of Polymer Science and Technology, Changchun Institute of Applied Chemistry, Chinese Academy of Sciences, Changchun 130022, China

**Keywords:** ionic liquid monomer, protein imprinting, accurate recognition, multi-anchoring effect

## Abstract

Protein molecularly imprinted polymers (MIPs), as artificial antibodies, are promising for protein separation due to their low cost, easy preparation, and high stability, but their performance is limited by poor mass transfer, imprecise imprinting, and single interaction modes. Herein, dendritic mesoporous silica nanoparticles (DMSNs) were used as the support, and a self-designed multifunctional poly(ionic liquid) macromonomer (p(VIMCD-co-VAIM-co-VSIM-co-VVIM)) served as the functional monomer to achieve directional anchoring of cytochrome C (Cyt-C). Surface-imprinted microspheres (DMSNs@MPS@PILs-MIPs) were prepared via free-radical copolymerization for Cyt-C recognition. The DMSNs possessed interconnected mesoporous channels, good dispersibility, an average particle size of ~80 nm, and a specific surface area of 267.97 m^2^/g. Ionic liquid monomers were synthesized via alkylation, and the macromonomer was constructed through a two-step method. Molecular dynamics simulations and spectroscopic characterization revealed the macromonomer-stabilized Cyt-C conformation, with interactions dominated by van der Waals forces. The DMSNs@MPS@PILs-MIPs featured a thin imprinted layer (~5 nm) to reduce mass-transfer resistance. Adsorption studies showed Cyt-C adsorption followed Langmuir and pseudo-second-order models, with a maximum capacity of 383.14 mg/g and an imprinting factor of 2.17. Only 12% capacity loss occurred after repeated cycles, indicating robust regeneration stability. This study provides a feasible strategy for constructing protein surface-imprinted polymers based on multifunctional synergistic interactions and conformational stabilization.

## 1. Introduction

Cytochrome C (Cyt-C) plays a pivotal role in biological systems, functioning as a key component of the mitochondrial electron transport chain during cellular respiration and serving as a crucial mediator in the initiation of apoptosis and related disease progression [1,2,3]. However, due to the intricate structure of Cyt-C and its susceptibility to conformational distortion under environmental fluctuations, achieving high-efficiency and specific recognition and separation of Cyt-C remains a formidable challenge in bioanalytical research [4,5]. Therefore, the development and rational design of molecularly imprinted polymers (MIPs)—referred to as “artificial antibodies” that serve as ideal biomimetic alternatives to natural receptors—have emerged as a promising solution [6]. Through imprinting polymerization and subsequent template elution, MIPs are fabricated with tailored imprinted cavities that are complementary to the target molecules in terms of shape, size, and functional groups, enabling the smart recognition of proteins via the classic lock-and-key mechanism [7,8]. However, traditional MIPs for macromolecules such as Cyt-C are frequently hindered by restricted mass transfer, “distortion” of imprinted cavities resulting from protein structural alterations, and the limited, single-mode interactions between protein surfaces and monomers [9,10,11,12].

Electroanalytical techniques have been widely applied to investigate the electrochemical behaviors of metalloproteins such as Cyt-C. Among these studies, Taniguchi and co-workers systematically explored the redox behavior of Cyt-C at electrode interfaces, providing important insights into protein electron-transfer mechanisms and the development of electrochemical protein sensors. However, achieving highly selective recognition and separation of Cyt-C in complex biological environments remains a major challenge [13,14]. To improve recognition efficiency, surface imprinting technology (SIT) confines imprinted sites to the material surface, thereby shortening the diffusion pathway of target molecules, reducing mass-transfer resistance, and enhancing the accessibility of recognition sites [15,16]. Compared with traditional bulk imprinting, which often suffers from difficult template removal and deeply buried binding sites, SIT exhibits significant advantages in protein recognition and has been widely employed for the selective recognition of Cyt-C [17,18]. Based on this strategy, Dechrirat et al. first combined epitope imprinting with electrochemical surface imprinting to directly fabricate ultrathin MIP films on gold transducer surfaces, improving both protein detection performance in aqueous media and interfacial electron-transfer efficiency [19]. Subsequently, researchers further optimized surface-imprinted systems through synergistic regulation of material structures and recognition mechanisms. For example, Emel et al. [20] constructed an ultrathin imprinted layer on bacterial cellulose nanofibers, significantly reducing mass-transfer resistance and accelerating binding kinetics. Qin et al. [21] combined epitope imprinting, metal chelation, and the interfacial synergistic effect of magnetic carbon nanotubes to realize the highly selective separation of Cyt-C in complex samples. Hence, the development of protein surface-imprinted materials by integrating surface imprinting technology facilitates Cyt-C mass transfer [21,22,23].

While surface imprinting technology significantly improves interfacial mass transfer and reduces nonspecific binding, the conformational stability of template proteins during polymerization remains a critical bottleneck limiting recognition precision. Ionic liquids (ILs) are organic molten salts composed of anions and cations that exist in a liquid state at room temperature. They possess multiple merits, including tunable anionic and cationic structures, high biological activity, excellent stability, and good solubility [24,25,26]. Studies have revealed that ILs elaborately designed with chaotropic cations and kosmotropic anions in accordance with the Hofmeister series [27,28] can better stabilize biomacromolecules. For instance, Hu’s group [29] employed an imidazolium-based amphiphilic ionic liquid as a surfactant to construct molecularly imprinted mesoporous materials for the selective recognition of Cyt-C. The resulting material exhibited a high specific surface area and an ordered mesoporous structure, delivering a high adsorption capacity (86.47 mg/g) and rapid adsorption kinetics (equilibrium reached within 20 min) toward Cyt-C. Moreover, Hu’s group has developed various IL-based functional monomers, cross-linkers, and emulsifiers to enhance the structural stability of template polypeptides and template proteins [29,30,31,32,33]. Song et al. [34] designed a specially structured block macromolecular functional monomer (MFM) containing both functional and cross-linking segments via RAFT polymerization and employed it to construct surface-imprinted BSA microspheres. Compared with the corresponding micromolecular functional monomer, the block MFM better preserved the native secondary structure of proteins and significantly enhanced the adsorption capacity and imprinting factor. Given the structural similarities across different proteins, this strategy can also be readily applied to Cyt-C systems. Therefore, the design of ILs macromonomers with large molecular size and low mobility to maintain the conformational integrity of protein molecules enables the accurate imprinting of proteins [35].

Moreover, the synergistic imprinting of proteins using functional monomers with diverse interaction modes can effectively address the challenges posed by the complex surface structures of proteins and the single-mode interaction between proteins and monomers. In existing studies, the metal coordination strategy exhibits an enhancement effect on protein imprinting recognition; the design and synthesis of multiple functional monomers for protein pre-immobilization have also improved the recognition performance of protein imprinting [36,37]. To mitigate the competition and interference of imprinting media with the interactions between target proteins and functional monomers, Du et al. [38] prepared a series of highly cross-linked raspberry-like spheres surface-functionalized with different ionic liquids. Their findings indicated that multiple interaction sites between Cyt-C and functional groups can strengthen their binding affinity, thereby enhancing the imprinting efficiency. Zhang et al. [39] exploited the structural characteristics of cyclodextrins (i.e., a hydrophobic cavity and a hydrophilic outer surface) to synthesize ionic liquid-*β*-cyclodextrin functional monomers, and further fabricated epitope magnetic imprinted nanospheres. These nanospheres achieve efficient recognition of Cyt-C via host–guest interactions to encapsulate target molecules. Thus, the construction of functional motifs capable of mediating multiple affinity interactions (e.g., electrostatic, hydrogen bonding, π-π, and hydrophobic interactions) and resisting nonspecific protein adsorption, to realize multiple anchoring and oriented imprinting, represents the most direct and effective approach to overcoming the bottleneck of low imprinting efficiency and poor selective recognition in protein imprinting [40,41].

Therefore, in this work, a series of ionic liquid monomers with anchoring sites was prepared via alkyl substitution reaction, and a poly(ionic liquid) (PIL) copolymeric macromonomer p(VIMCD-co-VAIM-co-VSIM-co-VVIM) was fabricated by free-radical copolymerization. The influence of the poly(ionic liquid) macromonomer on protein structural stability was systematically explored using advanced characterization techniques, including circular dichroism (CD) spectroscopy and synchronous fluorescence spectroscopy. Building upon this, protein surface-oriented imprinted microspheres (DMSNs@MPS@PILs-MIPs) were constructed by integrating a surface-directed imprinting strategy. Systematic investigations were conducted on the adsorption kinetics, adsorption thermodynamics, and recognition behaviors of the designed multi-anchoring protein-imprinted materials in both single-protein and multi-protein systems. This work aims to provide further insight into how multifunctional PIL macromonomers influence protein conformational preservation and interfacial recognition during protein imprinting, thereby offering a feasible strategy for the rational design of protein surface-imprinted materials.

## 2. Experimental Methods

### 2.1. Materials

All reagents used in the experiment, including N-vinylimidazole (VIM), bromoethane, benzyl chloride, 4-vinylbenzyl chloride (VBC), 2-chloroacetamide, β-cyclodextrin, 1-(phenylsulfonyl)-1H-imidazole, 1,3-propane sultone, 3-(trimethoxysilyl)propyl methacrylate (CTA), 2,2′-azobis(2-methylpropionitrile) (AIBN), ammonium persulfate (APS), N, N,N′,N′-tetramethylethylenediamine (TEMED), bovine hemoglobin (Hb), bovine serum albumin (BSA), and ovalbumin (OVA), were purchased from Aladdin Reagent Co., Ltd., Shanghai, China. Cetyltrimethylammonium bromide (CTAB), triethanolamine, sodium salicylate, tetraethyl orthosilicate, and cytochrome C (Cyt-C) were obtained from Macklin Biochemical Technology Co., Ltd., Shanghai, China. Lysozyme (Lyz) was purchased from Sigma-Aldrich., St. Louis, MO, USA. All solvents used in the experiment, such as sodium hydroxide, ethyl acetate, acetonitrile, dimethyl sulfoxide (DMSO), and tetrahydrofuran (THF), were supplied by Sinopharm Chemical Reagent Co., Ltd., Shanghai, China.

### 2.2. Design and Synthesis of Imidazolium-Based Ionic Liquid Monomers

**Figure 1:**
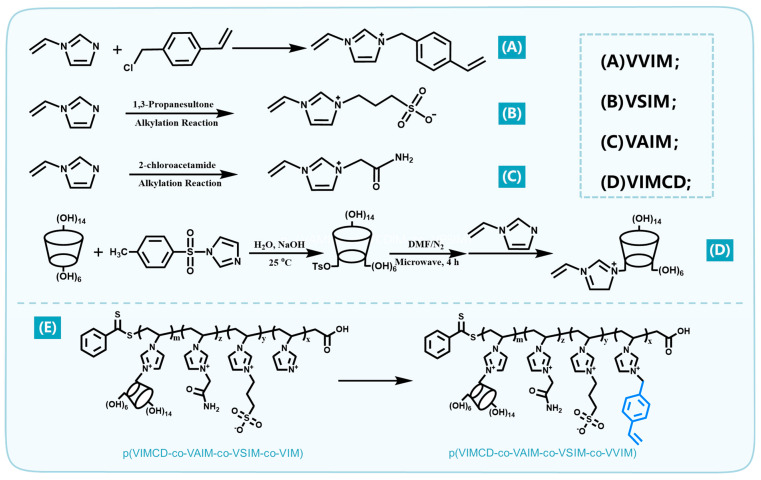
Schematic illustration of the synthesis of the 1-vinyl-3-(4-vinylbenzyl)imidazolium (VVIM) (**A**); 1-vinyl-3-sulfopropyl-imidazolium (VSIM) (**B**); 1-vinyl-3-acetamidoimidazolium (VAIM) (**C**); toluenesulfonated 1-vinylimidazole-*β*-cyclodextrin (VIMCD) (**D**); poly(ionic liquid) block copolymer macromonomers p(VIMCD-co-VAIM-co-VSIM-co-VVIM) (**E**).

Polymerizable ionic liquids, including 1-vinyl-3-(4-vinylbenzyl)imidazolium (VVIM), zwitterionic-structured 1-vinyl-3-sulfopropyl-imidazolium (VSIM), and 1-vinyl-3-acetamidoimidazolium (VAIM), and toluenesulfonated 1-vinylimidazole-*β*-cyclodextrin (VIMCD), were prepared via alkylation substitution reaction. The corresponding synthetic routes are illustrated in Figure 1A, Figure 1B, Figure 1C, and Figure 1D, respectively. Taking the synthesis of VAIM as a typical example, the acetamide group of 2-chloroacetamide substituted the hydrogen atom on the imidazole ring of N-vinylimidazole (VIM) to yield VAIM. The detailed synthetic procedure is described as follows: VIM (3.10 g, 33.00 mmol) and 2-chloroacetamide (2.90 g, 31.00 mmol) were accurately weighed and dissolved in ethanol solution. The mixture was mechanically stirred at room temperature for 24 h to proceed with the reaction. After the reaction was completed, the crude product was extracted with ethyl acetate, followed by lyophilization to obtain the target ionic liquid (VAIM). The detailed synthetic protocols for the other ionic liquids are provided in the Supporting Information. The chemical structure, purity, and properties of the as-prepared ionic liquids were characterized by nuclear magnetic resonance spectroscopy (^1^H NMR) and Fourier transform infrared spectroscopy (FT-IR).

### 2.3. Preparation of Poly(Ionic Liquid) Block Copolymer Macromonomers

CTA (70 mg, 0.33 mmol), VAIM (983 mg, 5.00 mmol), VIMCD (1289 mg, 1.00 mmol), VSIM (1081 mg, 5.00 mmol), VIM (470 mg, 5.00 mmol), and AIBN (22 mg, 0.13 mmol) were sequentially added into a Schlenk flask, followed by the addition of 40.00 mL of dimethyl sulfoxide (DMSO) to achieve complete dissolution. Under a nitrogen atmosphere, thermal-initiated polymerization was carried out at 70 °C for 16 h. Upon completion, the Schlenk flask was immediately immersed in liquid nitrogen to quench the polymerization. The reaction mixture was precipitated and washed with excess cold tetrahydrofuran (THF). The crude product was further purified by dialysis against deionized water (molecular weight cutoff, MWCO = 1500 Da) and then freeze-dried to obtain the precursor copolymer p(VIMCD-co-VAIM-co-VSIM-co-VIM), abbreviated as Macro-co-1. Subsequently, 1 g of the as-obtained precursor copolymer p(VIMCD-co-VAIM-co-VSIM-co-VIM) was dissolved in 40.00 mL of DMSO together with VBC (153 mg, 1 mmol) and a trace amount of hydroquinone. The reaction was conducted at 60 °C for 24 h under a N_2_ atmosphere. The crude product was precipitated and washed with excess cold THF three times, purified by dialysis against deionized water (MWCO = 1500 Da), and lyophilized to afford the final macromolecular functional monomer p(VIMCD-co-VAIM-co-VSIM-co-VVIM), abbreviated as Macro-co-2.

### 2.4. Effect of p(VIMCD-co-VAIM-co-VSIM-co-VVIM) on the Structural Stability of Proteins

#### 2.4.1. Spectroscopic Test of the Interaction Between p(VIMCD-co-VAIM-co-VSIM-co-VVIM) and Cyt-C

Aqueous solutions of poly(ionic liquid) block copolymer macromonomers p(VIMCD-co-VAIM-co-VSIM-co-VVIM) incubated with Cyt-C (0.05 mg/mL) for 24 h were used as the research objects, where the mass ratio of target protein to macromonomers was set to 1:0, 1:1, 1:2, 1:3, and 1:4, respectively. Circular dichroism (CD) spectroscopy was employed to investigate the changes in the secondary structure of Cyt-C in the far-UV region (200–250 nm), particularly the content of α-helical structures corresponding to the characteristic peaks at 208 nm and 222 nm. The contents of secondary structural elements (*α*-helix, *β*-sheet, *β*-turn, and random coil) of Cyt-C were calculated by fitting the CD spectra using the neural network algorithm (CDNN v2.1) provided by Applied Photophysics Ltd., Leatherhead, Surrey, UK. In addition, synchronous fluorescence spectroscopy was applied to record the spectral information of tyrosine (Δλ = 15 nm) and tryptophan amino acid residues (Δλ = 60 nm) in the protein within the wavelength range of 190–250 nm. The changes in the tertiary structure of Cyt-C were further evaluated by monitoring the variations in the microenvironment surrounding the intrinsic chromophores of the protein [42,43]. The interaction studies between VAIM, VCDIM, VSIM, VVIM, and Cyt-C were conducted following the same experimental procedures and characterization methods described above.

#### 2.4.2. Molecular Dynamics Simulation Study on the Interaction Mechanism Between p(VIMCD-co-VAIM-co-VSIM-co-VVIM) and Cyt-C

To gain insight into the interaction mechanism between p(VIMCD-co-VAIM-co-VSIM-co-VVIM) and Cyt-C, molecular dynamics simulations (MDs) were employed to investigate the effects of the synthesized macromonomers on the secondary structure, hydrogen bonds, and energy of the target protein Cyt-C. First, the geometric structure of the imidazolium-based ionic liquid macromonomer was constructed and preliminarily optimized using Chem3D 20.0. Rigid docking between the target protein (PDB ID: 5ty3) and the optimized macromonomer was performed via the HDOCK web server, providing a reasonable initial structure for subsequent molecular dynamics simulations. Second, the Gaussian 16 program was adopted to conduct preliminary geometric optimization of the poly(ionic liquid) block macromonomer, with the B3LYP/6-31G*(d) basis set and the Hartree-Fock method selected for calculation. Further optimization and electrostatic potential calculation were carried out sequentially, and the force field parameters of the above molecular structures were obtained using AmberTools 25. MDs were implemented with Gromacs 2021. Specifically, energy minimization of the simulation system was performed via the steepest descent method under the force convergence criterion of 1000 kJ/(mol·nm). Subsequently, NVT ensemble simulation (298.15 K, 100 ps) and NPT ensemble simulation (1 atm, 100 ps) were conducted successively, followed by a 50 ns molecular dynamics simulation. Relevant methods for the interaction between ionic liquid monomers and Cyt-C are shown in the Supporting Information.

### 2.5. Fabrication of DMSNs@MPA@PILs-MIPs Nanospheres

#### 2.5.1. Preparation of DMSNs@MPS

Triethanolamine, cetyltrimethylammonium bromide (CTAB), and sodium salicylate were dissolved in ultrapure water, followed by incubation at 80 °C in a water bath at a stirring speed of 400 rpm for 30 min. Subsequently, tetraethyl orthosilicate (TEOS) was added to the mixture, and the reaction was continued for another 60 min. After the reaction was completed, the resulting product was washed with ultrapure water for 2–3 times, dried, and then calcined at 600 °C for 8 h in an air atmosphere to obtain dendritic mesoporous silica nanoparticles (DMSNs). The as-prepared DMSNs were further calcined at 200 °C for 12 h, cooled to room temperature, and then dispersed in anhydrous toluene. The synthesized MPS reagent was added to the dispersion, and the mixture was subjected to a reflux reaction at 90 °C for 24 h. After centrifugation and washing, the product was cured for 1 h to afford MPS-modified dendritic mesoporous silica nanoparticles (DMSNs@MPS).

#### 2.5.2. Preparation of DMSNs@MPS@PILs-MIPs Surface-Oriented Imprinted Nanospheres

20.00 mg of DMSNs@MPS was weighed and ultrasonically dispersed in phosphate buffer solution (PBS). Then, 80.00 mg of the macromonomer p(VIMCD-co-VAIM-co-VSIM-co-VVIM) was added, followed by magnetic stirring for 30 min to obtain the prepolymer solution. The above solution was transferred to a 50 mL round-bottom flask, and 20.00 mg of Cyt-C was added into it. The mixture was incubated at 25 °C for 1 h to realize the full self-assembly of the target protein and macromonomer molecules, yielding a prepolymer solution with pre-immobilized target protein. Subsequently, 2,2′-azobisisobutyronitrile (AIBN, 5.00 mg, 0.04 mmol) was added to the resultant solution. After purging with nitrogen, the reaction was carried out for 24 h under magnetic stirring. The product obtained after centrifugation was washed with deionized water and 0.50 M NaCl solution repeatedly until no template molecules could be detected in the washing solution by ultraviolet (UV) spectroscopy. Finally, the product was freeze-dried to obtain the Cyt-C protein surface-oriented imprinted nanospheres (DMSNs@MPS@PILs-MIPs). The corresponding non-imprinted nanospheres (DMSNs@MPS@PILs-NIPs) were synthesized under the identical conditions to those of the imprinted nanospheres, with the only difference that the target protein Cyt-C was not added during the preparation process.

### 2.6. Characterization of Physicochemical Properties and Adsorption Performance Tests of DMSNs@MPS@PILs-MIPs Nanospheres

The purity and chemical structure of the synthesized ionic liquids were characterized by a nuclear magnetic resonance (^1^H NMR) spectrometer (BRUKER AVANCE 400, Bruker Corporation, Karlstuhe, Baden-Wurttemberg, Germany). For Fourier transform infrared spectroscopy (FT-IR) measurements, the sample to be tested was mixed with KBr and pressed into pellets, and the spectra were recorded in transmission mode using an FT-IR spectrometer (iS20, Thermo Fisher Scientific Inc., Waltham, MA, USA). The molecular weight of the poly(ionic liquid) macromonomer was determined by gel permeation chromatography (GPC) (Agilent 1260 Infinity II, Santa Clara, CA, USA).

The surface morphology and microstructure of the prepared nanospheres were observed by a field-emission scanning electron microscope (FESEM, Verios G4, FEI Inc., Hillsboro, OR, USA) and a high-resolution transmission electron microscope (HRTEM, S-TWIN, FEI Inc., Hillsboro, OR, USA). All SEM and TEM images were processed using ImageJ software (https://imagej.nih.gov/ij/, accessed on 20 May 2024) for improved presentation. The elemental changes during the grafting modification of the nanospheres were determined by an X-ray photoelectron spectroscopy (XPS) spectrometer (Thermo Kalpha, Thermo Fisher Scientific Inc., Waltham, MA, USA). The specific surface area and pore size of the materials were measured using a specific surface area and pore size analyzer (Micromeritics 3Flex, Micromeritics (Shanghai) Instrument Co., Norcross, GA, USA). The thermal stability and decomposition temperature of the core-shell structured materials were evaluated by a thermogravimetric analyzer (TGA, STA7200RV, Hitachi Ltd., Tokyo, Japan). The effects of ionic liquid monomers on the secondary and tertiary structures of proteins were investigated using a circular dichroism (CD) spectrometer (Chirascan, Applied Photophysics Ltd. Leatherhead, UK) and a fluorescence spectrometer (F-4500, Hitachi Ltd., Tokyo, Japan), respectively. According to the Bradford assay, the target protein content in the supernatant after adsorption by the imprinted nanospheres was determined using an ultraviolet-visible (UV-Vis) spectrophotometer (UV-2550, Shimadzu Corporation, Kyoto, Japan) at the characteristic absorption wavelengths of the target protein and competitive proteins. The selective recognition performance of the imprinted and non-imprinted nanospheres for the target protein was assessed by a sodium dodecyl sulfate-polyacrylamide gel electrophoresis (SDS-PAGE) apparatus (DYY-6C, Beijing Liuyi Instrument Factory, Beijing, China).

### 2.7. Adsorption Performance of DMSNs@MPS@PILs-MIPs Nanospheres

Accurately weigh 5.00 mg of DMSNs@MPS@PILs-MIPs/NIPs nanospheres, and ultrasonically disperse them in 10.00 mL of PBS buffer (pH 7.40, 10.00 mM) containing cytochrome C (Cyt-C) at a concentration of 1.00 mg/mL. Transfer the mixture into a constant temperature water bath shaker at a rotation speed of 200 rpm, incubate it at 25 °C for a certain period of time for adsorption, and then centrifuge the mixture. Collect the protein-containing supernatant and determine the protein concentration using an ultraviolet (UV) spectrophotometer. The adsorption capacity *Q*_e_ (mg/g) of DMSNs@MPS@PILs-MIPs/NIPs nanospheres for Cyt-C was calculated according to Formula (1).

(1) $$Q_{e}=(C_{0}-C_{e})V/m$$

where *C*_0_ represents the initial concentration of the Cyt-C solution (mg/mL); *C*_e_ represents the concentration of Cyt-C protein after adsorption (mg/mL); *V* represents the volume of the Cyt-C protein solution (mL); and m represents the mass of DMSNs@MPS@PILs-MIPs/NIPs nanospheres (g).

### 2.8. Selective Recognition Performance of DMSNs@MPS@PILs-MIPs Nanospheres

Lysozyme (Lyz), bovine serum albumin (BSA), ovalbumin (OVA), and bovine hemoglobin (Hb) were selected as competitive proteins to investigate the selective recognition performance of DMSNs@MPS@PILs-MIPs/NIPs toward the target protein via adsorption experiments. The detailed experimental procedures were as follows: 10.00 mg of DMSNs@MPS@PILs-MIPs/NIPs were accurately weighed and ultrasonically dispersed in 10.00 mL of PBS buffer (pH 7.40, 10.00 mM) containing a mixture of BSA, OVA, Hb and Cyt-C, with each protein at a concentration of 1.00 mg/mL. The mixture was incubated in a constant temperature water bath shaker at 200 rpm and 25 °C for 2 h for adsorption. After adsorption equilibrium was achieved, the mixture was centrifuged and the supernatant was collected, followed by sodium dodecyl sulfate-polyacrylamide gel electrophoresis (SDS-PAGE) analysis. For SDS-PAGE measurements, 12.5% polyacrylamide separating gel and 5% polyacrylamide stacking gel were used, with a concentrated sample loading volume of 10.00 µL.

Imprinting factor (*IF*) and selectivity factor (*β*) are important parameters for evaluating the selective recognition performance of imprinted nanospheres toward the target protein, which are calculated according to Formula (2) and Formula (3), respectively:

(2) $$IF={\;Q}_{\mathrm{MIPs}}/Q_{\mathrm{NIPs}}$$

where *Q*_MIPs_ is the adsorption capacity of DMSNs@MPS@PILs-MIPs nanospheres for the protein (mg/g); *Q*_NIPs_ is the adsorption capacity of DMSNs@MPS @PILs-NIPs (mg/g).

(3) $$\beta ={IF}_{\mathrm{template}}/{IF}_{\mathrm{ana}}$$

where ${IF}_{\mathrm{template}}$ is the imprinting factor of the imprinted nanospheres for the template protein Cyt-C; *IF*_ana_ is the imprinting factor of the imprinted nanospheres for the competitive proteins.

### 2.9. Regeneration Performance of DMSNs@MPS@PILs-MIPs

The target protein-imprinted materials can be recycled for reuse after elution and regeneration. Specifically, 10.00 mg of DMSNs@MPS@PILs-MIPs/NIPs nanospheres were accurately weighed and ultrasonically dispersed in 10.00 mL of PBS buffer solution (pH 7.40, 10.00 mM) containing Cyt-C at a concentration of 1.00 mg/mL. The mixture was then placed in a thermostatic water bath shaker at 25 °C. After reaching adsorption equilibrium, the mixture was centrifuged to separate and collect the supernatant. According to the Bradford method, the absorbance of the adsorbed solution was measured at 595 nm using a UV-visible spectrophotometer, and the adsorption capacity was further calculated. Subsequently, the adsorption-saturated nanospheres were transferred into a centrifuge tube, and a methanol-acetic acid mixture was added. The mixture was oscillated at a constant temperature until desorption equilibrium was achieved, followed by vacuum drying to obtain regenerated imprinted nanospheres. The regenerated nanospheres were subjected to the repeated “adsorption–separation–desorption” cycle, which was conducted for 5 consecutive times to evaluate the recycling performance.

## 3. Results and Discussion

### 3.1. Synthesis and Characterization of a Series of Imidazolium Ionic Liquids and Poly(Ionic Liquid) Macromonomers

**Figure 2:**
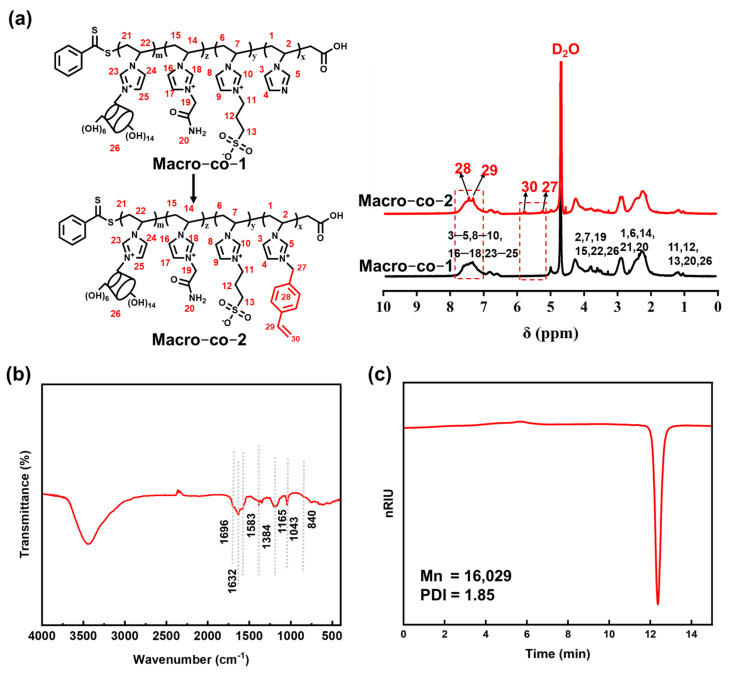
1H NMR spectrum of the poly(ionic liquid) macromonomer (**a**); FT-IR spectrum of the poly(ionic liquid) macromonomer (**b**); GPC profiles of the poly(ionic liquid) macromonomer (**c**).

The poly(ionic liquid) macromolecular monomer was synthesized via a two-step procedure, and the corresponding ^1^H NMR results are displayed in Figure 2a. For Macro-co-1, the signals at 7.31, 6.80, and 6.62 ppm are assigned to the characteristic protons on the imidazole ring. The peaks observed at 4.27, 3.84, 3.65, and 2.92 ppm correspond to the protons of hydroxyl groups and the adjacent methylene groups in the cyclodextrin structural unit, respectively. Moreover, the signals at 1.19 and 1.05 ppm are attributed to the protons of methylene groups connected to the amide group, hydroxyl groups in the cyclodextrin moiety, and the propane sulfonic acid segment. Compared with Macro-co-1, new characteristic peaks appear at 7.45, 7.34, 5.86, and 5.24 ppm in the spectrum of Macro-co-2, which are ascribed to the aromatic protons of the benzene ring and vinyl protons in the vinylbenzyl group. These results indicate that the vinylbenzyl group was successfully grafted onto Macro-co-1, confirming the successful synthesis of the macromolecular monomer Macro-co-2. The FT-IR characterization of the macromonomer Macro-co-2 is shown in Figure 2b. Characteristic peaks at 840 cm^−1^ and 1583 cm^−1^ are assigned to the out-of-plane bending vibration of C-H on the benzene ring and the skeletal stretching vibration of C=C in VVIM, respectively. The absorption peaks at 1696 cm^−1^ and 1632 cm^−1^ are attributed to the stretching vibration of the amide I band (C=O) and the bending vibration of the amide II band (N-H) in the primary amide of VAIM, respectively. The characteristic peak at 1165 cm^−1^ is ascribed to the symmetric stretching vibration of the S=O group in the sulfonic acid moiety of VSIM. Meanwhile, the absorption peaks at 3380 cm^−1^ and 1043 cm^−1^ correspond to the stretching vibrations of -OH and -C-O (bonded to hydroxyl groups) in the VIMCD structure, respectively, further confirming the successful synthesis of the macromonomer. Furthermore, the number-average molecular weight and molecular weight distribution of the macromolecular monomer were characterized by gel permeation chromatography (GPC), as presented in Figure 2c. As can be observed from Figure 2c, the relative molecular weight of the poly(ionic liquid) macromolecular monomer is approximately 16,029, with a polydispersity index (PDI) value of around 1.85.

### 3.2. Regulatory Mechanism of p(VIMCD-co-VAIM-co-VSIM-co-VVIM) on the Structural Stability of Proteins

**Figure 3:**
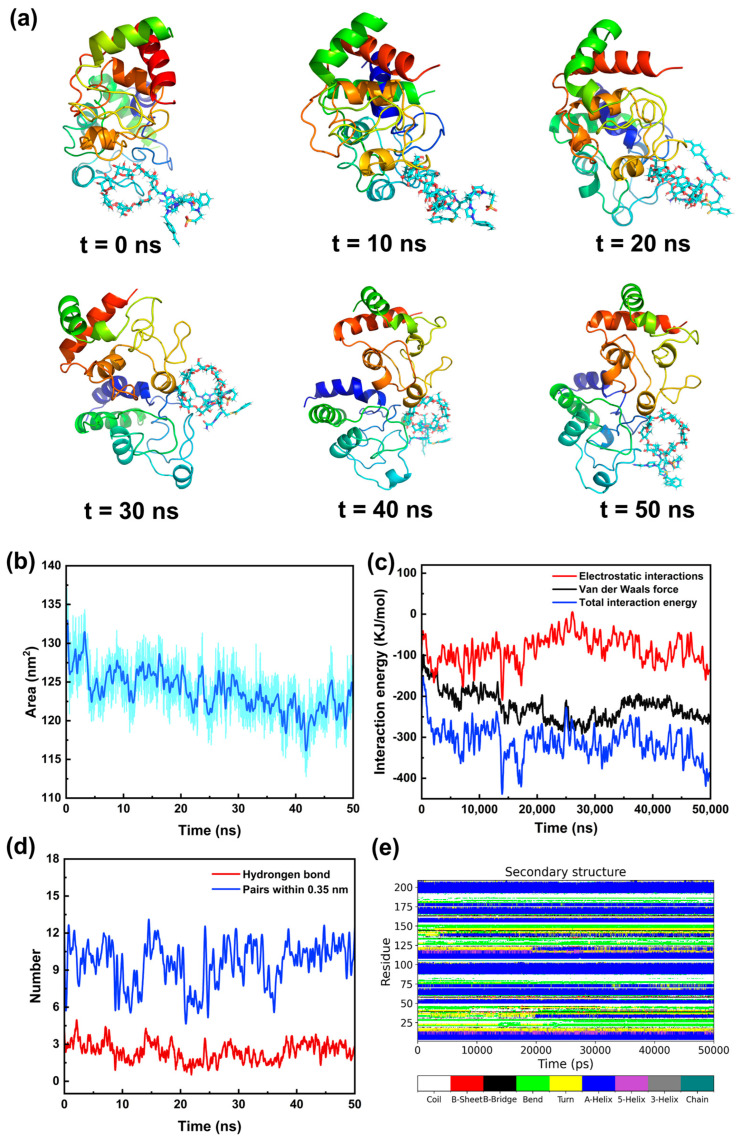
Conformational changes of the macromolecular monomer and cytochrome C (Cyt-C) at different simulation times (**a**), molecular dynamics simulation of the interaction process between the macromolecular monomer and Cyt-C: changes in solvent-accessible surface area (**b**), changes in interaction energy (**c**), changes in the number of hydrogen bonds (**d**), and the secondary structure evolution of Cyt-C (**e**).

We investigated the effects of the poly(ionic liquid) macromonomer on the conformational, secondary structural, energetic, and hydrogen-bonding variations of Cyt-C, so as to confirm whether the macromonomer can stabilize the protein structure for subsequent accurate imprinting. Figure 3a presents representative conformational snapshots of the p(VIMCD-co-VAIM-co-VSIM-co-VVIM)-Cyt-C complex at different simulation time points. The protein conformation reached an approximate equilibrium within 20 ns, and a 50 ns simulation duration was sufficient to form stable interactions between the protein and macromonomer. During the whole simulation period, the structure of Cyt-C did not depolymerize; although its conformation was slightly loosened, it remained stable and maintained a high proportion of α-helix. The solvent-accessible surface area (SASA) evolution of Cyt-C during molecular dynamics simulation is shown in Figure 3b. A slight reduction in SASA was observed at the initial stage, indicating structural adaptation of the protein upon binding with the polymer. Subsequently, the SASA values remained nearly constant throughout the simulation, suggesting that the overall protein conformation was well preserved without significant structural unfolding or compaction. Figure S3 shows the binding modes of four ionic liquid monomers with Cyt-C, where the monomers, key residues, and the interactions between monomers and Cyt-C are represented by stick, line and wireframe models, respectively. The simulation results demonstrate that electrostatic and hydrophobic interactions exert a prominent effect on the binding of ionic liquids to the protein. All four monomers interact and bind to the hydrophobic microdomain of Cyt-C formed by VAL, LEU and NLE residues, which is a typical manifestation of hydrophobic interaction. In addition, a hydrogen bond interaction (indicated by green dashed lines) is observed between VSIM and ASN residues. In contrast, Figure 3c shows that the electrostatic interaction energy, van der Waals interaction energy and total interaction energy of the p(VIMCD-co-VAIM-co-VSIM-co-VVIM)-Cyt-C complex at equilibrium are −120.86 kJ/mol, −258.44 kJ/mol and −379.30 kJ/mol, respectively. These molecular dynamics simulation results of the macromolecular monomer-Cyt-C system confirm its multi-interaction synergistic recognition mechanism: the non-covalent recognition between them is dominated by short-range interactions (hydrogen bonds and van der Waals forces), with electrostatic forces providing auxiliary stability for the binding system. Moreover, the average number of hydrogen bonds between p(VIMCD-co-VAIM-co-VSIM-co-VVIM) and Cyt-C remained relatively stable during the entire simulation process (Figure 3d). This consistent hydrogen-bonding behavior further confirms that the protein structure maintained conformational stability upon complex formation. Figure 3e shows the full time evolution of the secondary structure of Cyt-C during its interaction with p(VIMCD-co-VAIM-co-VSIM-co-VVIM). It can be observed that after 50 ns of simulation, the proportion of *α*-helical structure in Cyt-C exhibited almost no change under the action of p(VIMCD-co-VAIM-co-VSIM-co-VVIM), which exhibits a significant stabilizing effect on the α-helical structure of Cyt-C.

**Figure 4:**
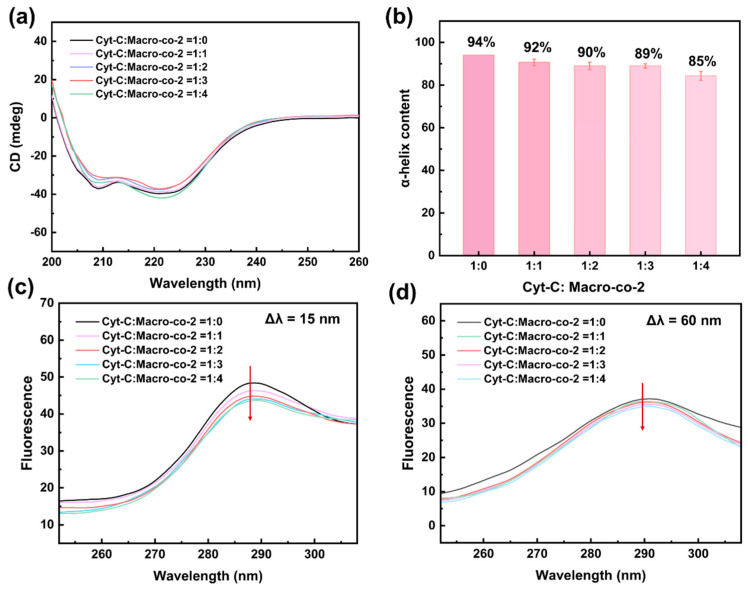
Effects of different doses of Macro-co-2 on the secondary structure of Cyt-C (**a**), changes in α-helix content in the secondary structure (**b**), and the tertiary structure (**c**,**d**).

Circular dichroism (CD) spectroscopy was used to analyze the effects of small-molecule monomers and macromonomers on the secondary structure of the protein. Compared with small-molecule monomers (detailed analysis is provided in the Supporting Information and Figure S4), the macromonomer can maintain the native conformation of Cyt-C relatively well. As shown in Figure 4a, when the mass ratio of Cyt-C to macromonomer reaches 1:4, the two typical negative peaks of Cyt-C at 208 nm and 220 nm remain intact with virtually unchanged peak positions, indicating that the secondary structure of Cyt-C was not significantly disrupted under the action of the synthesized macromonomer. This result was further verified by the changes in *α*-helix content calculated via CDNN (Figure 4b).

Synchronous fluorescence spectroscopy was used to obtain the information of tyrosine residues (Tyr, Δλ = 15 nm) and tryptophan residues (Trp, Δλ = 60 nm) in the protein, and the changes in the tertiary structure of the protein were investigated based on the microenvironmental variations around the intrinsic chromophores of the protein. Compared with small-molecule monomers (detailed analysis is provided in the Supporting Information and Figure S4), Figure 4c,d show that the introduction of the poly(ionic liquid) macromonomer caused concentration-dependent fluorescence quenching of intrinsic tryptophan and tyrosine residues in Cyt-C. This confirms the existence of specific interactions between the poly(ionic liquid) macromonomer and the protein, as well as slight local conformational changes in the microenvironment of the protein.

In summary, the synthesized macromolecular functional monomer had no significant effect on the secondary structure of Cyt-C and only induced slight microchanges in its tertiary structure, which was highly consistent with the conclusions of molecular dynamics simulations. This multifunctional macromonomer effectively overcame the destructive effects on the protein structure caused by the mixing of traditional small-molecule monomers. This demonstrates that the functional monomer possesses good biocompatibility and can interact with the target protein without destroying its native conformation, thereby making it suitable for the recognition and selective adsorption of the target protein in the subsequent preparation of molecularly imprinted polymers.

### 3.3. Preparation and Physicochemical Characterization of DMSNs@MPS @PILs-MIPs

**Figure 5:**
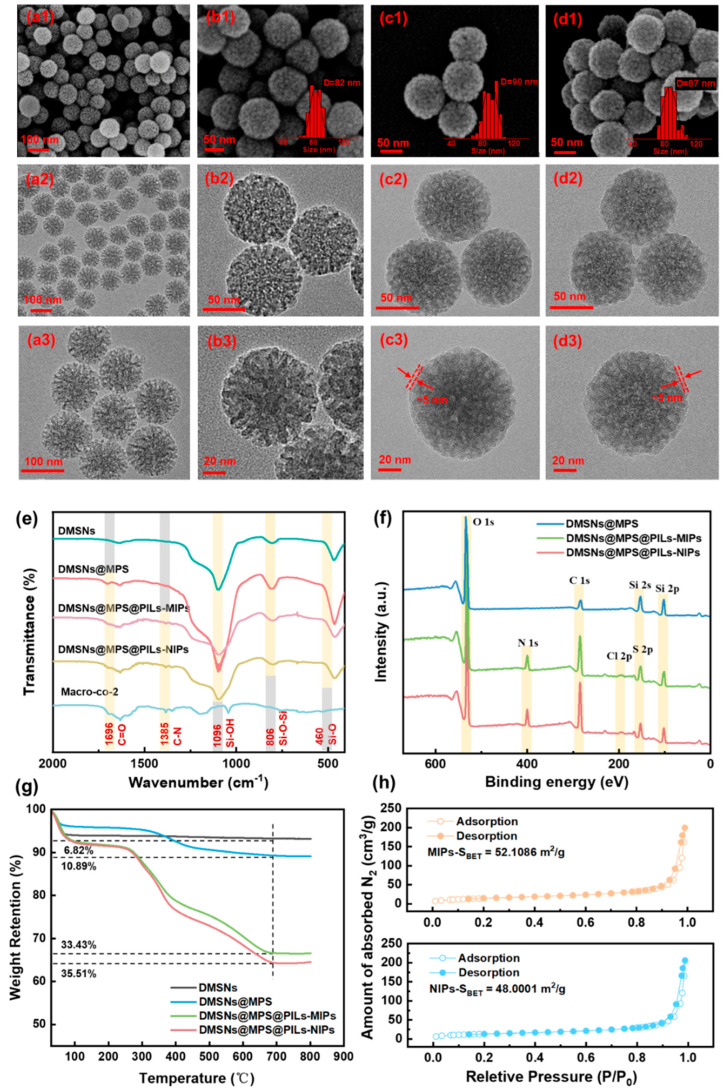
SEM (**a1**,**b1**) and TEM images (**a2**,**a3**,**b2**,**b3**) of DMSNs; SEM (**c1**) and TEM images (**c2**,**c3**) of DMSNs@MPS@PILs-MIPs, and SEM (**d1**) and TEM images (**d2**,**d3**) of DMSNs@MPS@PILs-NIPs; FT-IR spectra (**e**), XPS spectra (**f**), TGA curves (**g**), and nitrogen adsorption–desorption isotherms (**h**) of DMSNs@MPS@PILs-MIPs and DMSNs@MPS@PILs-NIPs.

The surface morphologies and structural features of DMSNs, DMSNs@MPS@PILs-MIPs, and DMSNs@MPS@PILs-NIPs nanospheres were characterized by scanning electron microscopy (SEM) and transmission electron microscopy (TEM), as shown in Figure 5(a1–d3). As observed in Figure 5(a1,b1), the dendritic silica nanoparticles exhibit abundant wrinkled structures on the surface, maintain excellent aqueous monodispersity, and possess uniform particle sizes with a diameter of approximately 80 nm. TEM images (Figure 5(a2,b2)) reveal that the introduction of the structure-directing agents sodium salicylate (NaSal) and cetyltrimethylammonium bromide (CTAB) endows the silica nanoparticles with radial, radially interconnected mesoporous channels extending from the particle center to the surface. The channels feature dendritic branching, regular arrangement, and uniform orientation. Accordingly, DMSNs@MPS@PILs-MIPs nanospheres were fabricated using Cyt-C as the template protein and p(VIMCD-co-VAIM-co-VSIM-co-VVIM) as the functional monomer. As shown in Figure 5(c1–c3), the nanospheres modified with the polymer shell still retain the radial channels and network structure of pristine DMSNs, with a thin polymer shell thickness of approximately 5 nm. The shell thickness of DMSNs@MPS@PILs-NIPs nanospheres (Figure 5(d1–d3)) is comparable to that of the imprinted nanospheres. The chemical structures of DMSNs, DMSNs@MPS@PILs-MIPs, and DMSNs@MPS@PILs-NIPs were characterized by Fourier transform infrared (FT-IR) spectroscopy, as presented in Figure 5e. The characteristic absorption peaks at 460 cm^−1^ (Si-O), 806 cm^−1^ (Si-OH), 1099 cm^−1^ (Si-O-Si), and 1696 cm^−1^ (C-O) confirm the existence of DMSNs and the successful modification of DMSNs@MPS. The characteristic peak at 1385 cm^−1^ (ascribed to the deformation and stretching vibrations of the imidazole ring) verifies the successful synthesis of DMSNs@MPS@PILs-MIPs and DMSNs@MPS@PILs-NIPs. X-ray photoelectron spectroscopy (XPS) results (Figure 5f) reveal that the characteristic peaks at 399.6 eV (N 1s), 198.7 eV (Cl 2p), 229.1 eV (S 2s), and 164.0 eV (S 2p) further confirm the presence of the polymeric monomers in DMSNs@MPS@PILs-MIPs and DMSNs@MPS@PILs-NIPs. Combined with thermogravimetric analysis (TGA, Figure 5g), the thermal degradation mass of the nanospheres follows the order: DMSNs < DMSNs@MPS < DMSNs@MPS@ PILs-MIPs < DMSNs@MPS@PILs-NIPs. Compared with DMSNs, the weight losses of the imprinted and non-imprinted nanospheres are 33.43% and 35.51%, respectively, which cross-validates the successful coating of the polymer shell. Furthermore, the pore structure and properties of the imprinted and non-imprinted nanospheres were investigated by N_2_ adsorption–desorption measurements, as presented in Figure 5h. Pristine DMSNs possess a high specific surface area of 267.9679 m^2^/g (Figure S5). After being coated with the poly(ionic liquid) imprinted layer, the specific surface area of the material decreases significantly. The calculated specific surface areas (SSAs) of DMSNs@MPS@PILs-MIPs and DMSNs@MPS@PILs-NIPs are 52.1086 m^2^/g and 48.0001 m^2^/g, respectively, demonstrating that the polymer shell has been successfully deposited on the surface of DMSNs and partially occupies the pore channels. The pore size distribution results (Figure S6) reveal that both imprinted and non-imprinted nanospheres maintain mesoporous characteristics; however, their pore volumes are much lower than that of pristine DMSNs, further confirming the successful formation of the polymer shell. Overall, it can be confirmed that DMSNs, DMSNs@MPS, DMSNs@MPS@PILs-MIPs, and DMSNs@MPS@PILs-NIPs have been successfully synthesized, and their high specific surface areas are beneficial for the subsequent adsorption and separation processes.

### 3.4. Investigation on the Adsorption Performance of DMSNsNs@MPS@ PILs-MIPs

**Figure 6:**
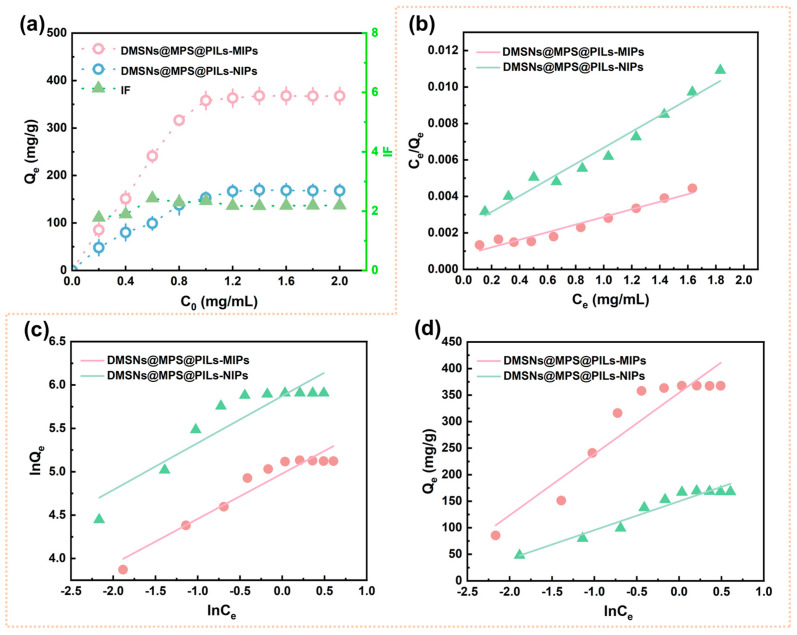
Adsorption isotherms at 25 °C of Cyt-C on DMSNs@MPS@PILs-MIPs and DMSNs@MPS@PILs-NIPs (**a**), Langmuir model (**b**), Freundlich model (**c**), and Temkin model (**d**).

The adsorption performances of DMSNs@MPS@PILs-MIPs and DMSNs@MPS@PILs-NIPs were investigated at 25 °C, and the corresponding results are displayed in Figure 6. The equilibrium adsorption isotherms of the imprinted microspheres were determined at Cyt-C equilibrium concentrations (*C*_e_) ranging from 0.00 to 2.00 mg/mL (Figure 6a). The adsorption capacity of DMSNs@MPS@PILs-MIPs for Cyt-C increases rapidly within the *C*_e_ range of 0.00–0.80 mg/mL and then gradually slows down, with adsorption equilibrium achieved at a Cyt-C concentration of 1.00 mg/mL. This phenomenon can be attributed to the gradual occupation of surface and internal recognition cavities by target protein molecules with increasing Cyt-C concentration until saturation is reached. Meanwhile, the reduced accessibility of Cyt-C to the imprinted cavities during adsorption further gives rise to the above variation in adsorption tendency. In contrast, DMSNs@MPS@PILs-NIPs reaches adsorption equilibrium at a Cyt-C concentration of 0.80 mg/mL, which arises from the nonspecific adsorption of proteins on non-imprinted microspheres. The adsorption isotherm data were evaluated using the Langmuir and Freundlich and Temkin models, and the corresponding linearized parameters are listed in Table S1. As revealed by isothermal adsorption fitting results (Table 1), the Langmuir isotherm model presents a correlation coefficient (*R*^2^) much closer to 1.00 than the Freundlich model and Temkin isotherm models, suggesting that the Langmuir model is more appropriate to describe the adsorption behavior of the as-prepared material. This also demonstrates that uniform imprinted cavities are distributed on the surface and in the interior of DMSNs@MPS@PILs-MIPs, and the adsorption of target protein obeys a monolayer adsorption mechanism [44]. In addition, the *K*_d_ value of DMSNs@MPS@PILs-MIPs (3.0900 × 10^−5^ mol/L) is lower than that of DMSNs@MPS@PILs-NIPs (4.1600 × 10^−5^ mol/L), which suggests a relatively stronger binding affinity for Cyt-C. This enhancement is attributed to the sufficient recognition cavities generated on the surface of the MIPs.

**Figure 7:**
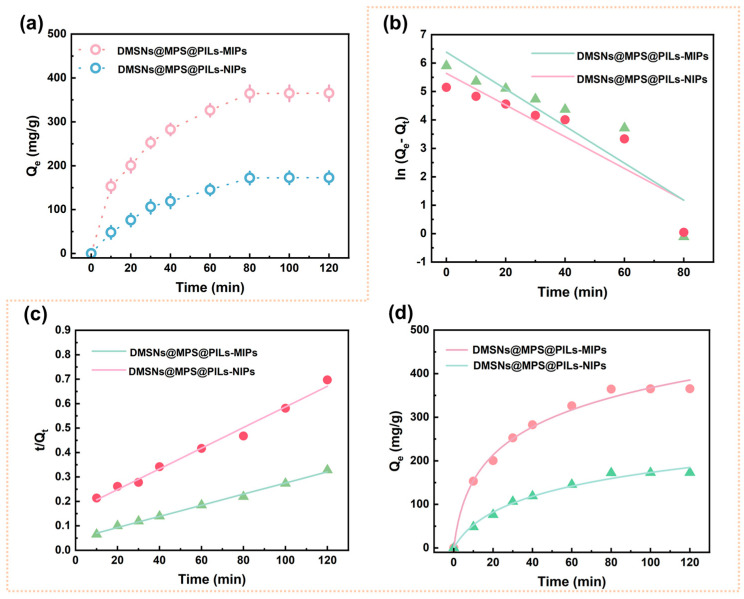
Kinetic adsorption profiles at 25 °C of Cyt-C on DMSNs@MPS@PILs-MIPs and DMSNs@MPS@PILs-NIPs (**a**), PFO model (**b**), PSO model (**c**), and Elovich model (**d**).

The adsorption kinetics of DMSNs@MPS@PILs-MIPs and DMSNs@MPS@ PILs-NIPs microspheres are displayed in Figure 7a. A rapid adsorption rate of Cyt-C toward DMSNs@MPS@PILs-MIPs is observed within 0~30 min, owing to the low mass-transfer resistance and favorable accessibility of Cyt-C to the imprinted cavities at the initial adsorption stage. The adsorption rate gradually decreases as the imprinted cavities are progressively occupied by Cyt-C molecules, accompanied by the increased steric hindrance of Cyt-C. Adsorption equilibrium is achieved at 80 min, with the adsorption rate approaching nearly zero. DMSNs@MPS@PILs-NIPs microsphere shows a similar kinetic trend to DMSNs@MPS@PILs-MIPs. However, no imprinted cavities matching the target protein are present in NIPs, and only nonspecific adsorption occurs, leading to a lower saturated adsorption capacity. Based on the above results, a Cyt-C equilibrium concentration of 1.00 mg/mL and an adsorption time of 80 min are adopted in the subsequent experiments.

To further evaluate the adsorption mechanism, the adsorption kinetics were fitted using the pseudo-first-order (PFO), pseudo-second-order (PSO) kinetic models, and the Elovich model, and the corresponding linearized parameters are listed in Table S2. The corresponding fitting curves are shown in Figure 7b–d, with the related parameters summarized in Table 2. As indicated in Table 2, the PSO kinetic model provides a better fit to the experimental adsorption data of both DMSNs@MPS@PILs-MIPs and DMSNs@MPS@PILs-NIPs, exhibiting higher correlation coefficients (*R*^2^ > 0.98) than the PFO model. These results suggest that the adsorption of Cyt-C on DMSNs@MPS@PILs-MIPs and DMSNs@MPS@PILs-NIPs is predominantly governed by a chemisorption mechanism. Furthermore, the Elovich model was also used for solid–liquid chemical adsorption to describe the heterogeneous diffusion process. In the Elovich model, the imprinted materials also exhibit a high correlation coefficient (*R*^2^ > 0.98), indicating that there is chemical adsorption between DMSNs@MPS@PILs-MIPs, DMSNs@MPS@PILs-NIPs and Cyt-C in the solution [45]. This is mainly due to the fact that the macromolecular monomer chains in DMSNs@MPS@PILs-MIPs are rich in active sites such as ionic bonds, hydrogen bonds and electrostatic interactions, which have the dual ability to form multiple non-covalent bonds with Cyt-C and recognize Cyt-C molecules through the complementary shape of Cyt-C on the cavity surface. These results indicate that the adsorption of Cyt-C on DMSNs@MPS@PILs-MIPs and DMSNs@MPS@PILs-NIPs is mainly controlled by the chemical adsorption mechanism.

### 3.5. Investigation on the Selective Recognition Performance of DMSNs@MPS@ PILs-MIPs

**Figure 8:**
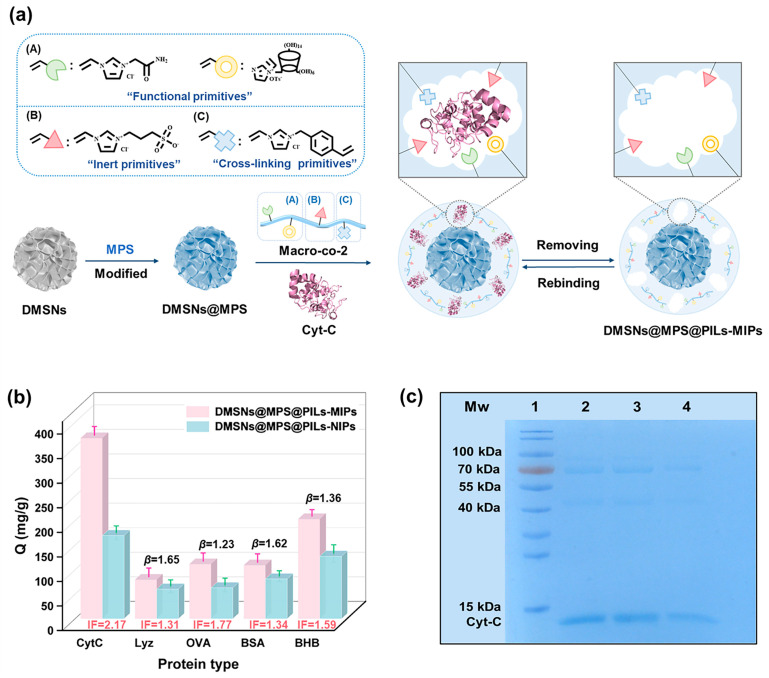
Scheme of synthesis and recognition performances of DMSNs@MPS@PILs-MIPs (**a**): Functional primitives (**A**); Inert Primitives (**B**); Cross-linking Primitives (**C**); Specific recognition adsorption (**b**) and SDS-PAGE analysis of adsorption of Cyt C by MIPs/NIPs with protein mixture (**c**): lane 1, protein molecular weight marker; lane 2, the protein mixture solution before adsorption; lane 3, proteins eluted with acetic acid (6.00 vt%) after adsorption by DMSNs@MPS@PILs-MIPs; lane 4, proteins eluted with acetic acid (6.00 vt%) after adsorption by DMSNs@MPS@PILs-NIPs.

According to the molecular weight (MW) and isoelectric point (pI) of Cyt-C (MW 13.20 kDa, pI 10.00), several competing proteins were selected to investigate the selective adsorption performance of the imprinted microspheres quantitatively and qualitatively, including Lyz (MW 14.40 kDa, pI 10.80), OVA (MW 43.00 kDa, pI 4.70), BSA (MW 66.20 kDa, pI 4.90), and BHB (MW 66.20 kDa, pI 4.90). The experimental results are displayed in Figure 8b. Obviously, the adsorption capacity of DMSNs@MPS@PILs-MIPs toward Cyt-C reaches 383.14 mg/g with a high imprinting factor (*IF*) of 2.17, indicating the optimal selective recognition performance. For Lyz with similar MW and pI values, *IF*_Lyz_ = 1.31 and the corresponding *β*_Lyz_ = 1.65. This is attributed to the fact that the multiple protein anchoring groups formed in the molecularly imprinted layer constructed by the poly(ionic liquid) macromonomer can only target Cyt-C (Figure 8a) rather than capture the smaller Lyz molecules. For OVA with a higher MW and lower pI, the dual effects of size difference and charge property lead to the worst adsorption selectivity of the imprinted material toward OVA (*IF*_OVA_ = 1.31, corresponding *β*_OVA_ = 1.65). Similarly, DMSNs@MPS@PILs-MIPs cannot effectively bind BSA and BHB either. Moreover, the adsorption capacities of DMSNs@MPS@PILs-NIPs toward Lyz, OVA, BSA, and BHB, as well as those of DMSNs@MPS@PILs-MIPs toward the above competing proteins, are all relatively low, suggesting that nonspecific adsorption plays a dominant role in these processes.

Subsequently, SDS-PAGE was utilized for the qualitative analysis of the selective adsorption performance of the imprinted materials, with the results shown in Figure 8c. Lane 1 represents the protein molecular weight marker; Lane 2 is the mixed protein solution of Cyt-C, OVA, BSA, and BHB before adsorption; Lane 3 is the protein solution from the first elution after adsorption by the imprinted microspheres; and Lane 4 is the protein solution from the first elution after adsorption by the non-imprinted microspheres. As observed from the protein bands, DMSNs@MPS@PILs-MIPs exhibited relatively higher adsorption toward Cyt-C than DMSNs@MPS@PILs-NIPs. In contrast, only limited differences were observed between the imprinted and non-imprinted microspheres in the adsorption of the other three competing proteins, suggesting that the adsorption of these proteins was mainly associated with nonspecific interactions.

In conclusion, both qualitative and quantitative competitive adsorption experiments indicate that the multiple-interaction structural and functional groups constructed on DMSNs@MPS@PILs-MIPs play an important role in the selective binding of Cyt-C, contributing to its recognition in mixed protein solutions.

### 3.6. Investigation on the Regenerability and Reusability of DMSNs@MPS@ PILs-MIPs

**Figure 9:**
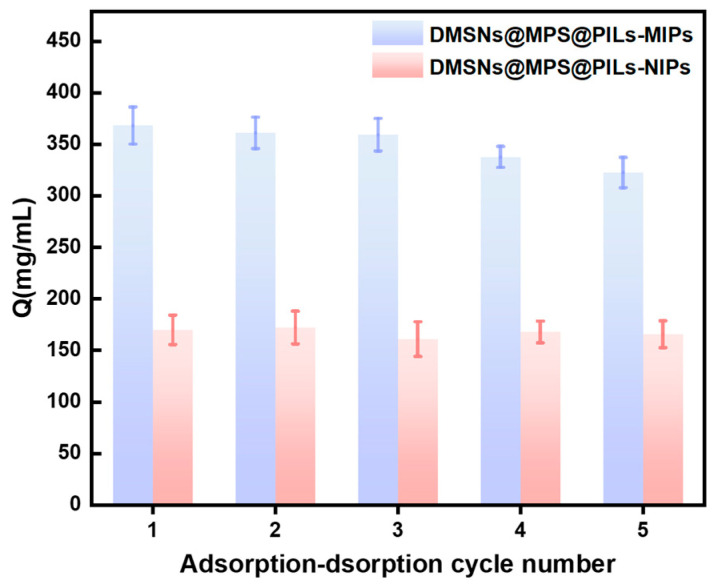
Adsorption–desorption cycle profiles of DMSNs@MPS@PILs-MIPs and DMSNs@MPS@PILs-NIPs.

Regenerability is a crucial index for evaluating the practical application value of imprinted materials. Figure 9 depicts the reusability of DMSNs@MPS@PILs-MIPs/NIPs nanospheres after five adsorption–desorption cycles. As shown in Figure 9, the adsorption capacity of DMSNs@MPS@PILs-MIPs nanospheres toward the target protein Cyt-C decreases slightly after each cycle. After five adsorption–desorption cycles, the adsorption capacity decreases from 367.6190 mg/g in the first cycle to 322.1138 mg/g in the fifth cycle, corresponding to an approximate loss rate of 12%, indicating acceptable reusability of the imprinted materials. In contrast, DMSNs@MPS@PILs-NIPs nanospheres exhibit almost unchanged adsorption performance during the repeated adsorption–elution processes. The adsorption capacity only decreases slightly from 169.3873 mg/g to 165.1853 mg/g after five cycles, retaining about 98% of the initial adsorption capacity and demonstrating a negligible loss in adsorption performance. This phenomenon can be attributed to the fact that partial imprinted cavities and binding sites of DMSNs@MPS@PILs-MIPs nanospheres are lost during recycling due to incomplete elution, resulting in the failure to rebind the target protein selectively. However, the protein anchoring sites within the imprinted cavities formed by the rigid polymer synthesized in this work are stable, and the multiple interactions between the polymer and the protein are not weakened, leading to a low loss of specific adsorption capacity.

### 3.7. Performance Evaluation of DMSNs@MPS@PILs-MIP

In recent years, significant efforts have been devoted to the separation of Cyt-C using MIT, with continuous optimization of material design to improve adsorption performance (Table 3). Canpolat et al. [46] developed an integrated imprinting system using lanthanide-coordinated cryogel technology with (MAAP)2-Ce(III) as the functional monomer, achieving a capacity of 98.33 mg/g and an *IF* of 4.46. While metal coordination enhances template–monomer interactions, the bulk cryogel structure still imposes mass-transfer limitations. Li et al. [29] utilized γ-CD host–guest interactions to construct SiO_2_-based epitope imprinted materials (*Q*_e_ = 86.47 mg/g, *IF* = 3.38), improving kinetics by increasing surface site exposure; Zhang et al. [47] reported similar findings, yet capacities remained relatively low, likely due to uneven site distribution on the silica surface. Mesoporous silica, with its uniform pore distribution and high permeability, offers a solution. For instance, Cheng et al. [48] achieved significant gains in capacity and selectivity (*Q*_e_ = 249.60 mg/g, *IF* = 3.80) by incorporating zwitterionic liquids into mesoporous SiO_2_. Qian et al. [49] constructed surface-imprinted nanoparticles based on CN@UIO-66 composites, reaching an ultra-high capacity of 815.00 mg/g and an *IF* of 6.10. Fan et al. [50] prepared calcium alginate nanofiber membranes via electrospinning (*Q*_e_ = 3312.00 mg/g), though the *IF* (1.66) suggested room for improvement in selectivity.

In comparison, the DMSNs@MPS@PILs-MIPs prepared in this study exhibited an adsorption capacity of 367.86 mg/g, which is at a relatively high level among the current literature. This is attributed to the high surface area and open pore structure of the dendritic DMSNs, as well as the synergistic effect of multiple interactions between the poly(ionic liquid) segments and Cyt-C. However, the imprinting factor of the as-prepared material was 2.17, indicating that the selectivity still requires further improvement. This may be attributed to the multi-site interaction characteristics of the multifunctional poly(ionic liquid) macromonomer. While the introduced synergistic weak interactions help stabilize the native conformation of Cyt-C, they may also cause weak nonspecific interactions with non-target proteins, thereby limiting the adsorption difference between MIPs and NIPs as well as the improvement in selectivity. Similar behavior has also been reported in the work of Fan et al., where materials with relatively high adsorption capacity simultaneously exhibited comparatively low imprinting factors [50]. Nevertheless, the present results suggest that multifunctional synergistic interactions are beneficial for constructing a mild protein-recognition interface with potential selective separation capability. Future work will focus on reducing nonspecific adsorption and further improving the selective recognition performance of the materials.

In summary, the obtained DMSNs@MPS@PILs-MIPs provide a feasible strategy for the selective separation and purification of Cyt-C based on multifunctional synergistic interactions and conformational stabilization. The prepared materials exhibit mild preparation and recognition conditions, low sample consumption, and potential selective recognition capability toward target proteins. More importantly, this work emphasizes the role of protein conformational stabilization in protein imprinting, which may offer new insights into the design of protein-imprinted materials with improved selective recognition performance. Future research will further focus on optimizing the comprehensive properties and practical applicability of the prepared materials:

1. The prepared DMSNs@MPS@PILs-MIPs will be applied to the analysis of actual samples to expand their practical application scenarios.
2. Poly(ionic liquid) macromonomer with multi-responsive characteristics (e.g., pH, temperature, and light responsiveness) will be further designed to achieve the controllable adsorption and release of target proteins and optimize the comprehensive performance of materials.
3. The structural design of functional groups will be directionally optimized to effectively reduce the cross-reactivity of structurally similar proteins, further strengthen the recognition specificity of materials, and provide theoretical and technical support for the development and practical application of high-performance protein-imprinted materials.

## 4. Conclusions

In this study, we successfully constructed a high-performance protein surface molecularly imprinted system to address the inherent limitations of traditional protein-imprinted materials, such as poor mass transfer, inaccurate imprinting, and single-mode protein–monomer interaction. Dendritic mesoporous silica nanoparticles (DMSNs) were selected as the support, and a self-designed poly(ionic liquid) macromonomer (p(VIMCD-co-VAIM-co-VSIM-co-VVIM), Macro-co-2) with multiple functional segments was used as the functional monomer to achieve directional anchoring of the template protein cytochrome C (Cyt-C). Protein molecularly imprinted microspheres (DMSNs@MPS@PILs-MIPs) were fabricated via free-radical copolymerization, realizing precise imprinting and specific recognition of Cyt-C. The as-prepared DMSNs exhibited radially oriented and interconnected mesoporous channels from the core to the surface, excellent aqueous monodispersity, an average particle size of approximately 80 nm, and a specific surface area of 267.9679 m^2^/g, providing an ideal support for the preparation of imprinted microspheres. A series of ionic liquid monomers was synthesized by alkylation reaction, and the poly(ionic liquid) macromonomer was further obtained through a two-step strategy. Combined with molecular dynamics simulations and spectroscopic characterization, we systematically clarified that the macromonomer could effectively stabilize the structure and conformation of Cyt-C, and the interaction between them was dominated by van der Waals forces, which laid a solid theoretical foundation for the accurate imprinting of Cyt-C during the pre-immobilization process. The prepared DMSNs@MPS@PILs-MIPs possessed a thin imprinted layer (≈5 nm), which contributed to reduced diffusion resistance and relatively fast adsorption behavior. Adsorption kinetic and thermodynamic experiments confirmed that the adsorption behavior of DMSNs@MPS@PILs-MIPs toward Cyt-C was well fitted by the Langmuir isotherm model and pseudo-second-order kinetic model, corresponding to typical monolayer chemisorption. Selective adsorption experiments demonstrated that the imprinted microspheres exhibited preferential recognition toward Cyt-C in both single-protein and competitive protein systems. Under competitive adsorption conditions, the adsorption capacity toward Cyt-C reached 383.14 mg/g with an imprinting factor of 2.17. Although a certain degree of cross-reactivity toward competing proteins was still observed, the incorporation of multiple interaction sites within the poly(ionic liquid) framework contributed to enhanced binding preference toward the target protein compared with the non-imprinted counterpart. In addition, the materials retained most of their adsorption capacity after repeated adsorption–desorption cycles, indicating acceptable regeneration stability. Overall, the present work demonstrates that integrating DMSNs with multifunctional poly(ionic liquid) macromonomers is a feasible strategy for constructing protein surface-imprinted materials with improved adsorption characteristics and selective recognition ability. Although further optimization is still needed to enhance the imprinting factor and specific recognition performance, this study provides useful experimental and theoretical insights for the design of protein-imprinted materials for protein-recognition and separation applications.

## Figures and Tables

**Table 1:** Parameters of the isotherm adsorption model fitted for cytochrome C (Cyt-C) adsorption.

Models		DMSNs@MPS@PILs-MIPs	DMSNs@MPS@PILs-NIPs
Langmuir	*Q*_m_	478.4688	227.2727
	*K*_d_	3.0900 × 10^−5^	4.1600 × 10^−5^
	*R* ^2^	0.9448	0.9645
Freundlich	1/*n*	0.5411	0.5222
	*K*_F_	354.25	144.03
	*R* ^2^	0.8299	0.9175
Temkin	A	21.5419	15.8000
	B	115.2567	54.3094
	*R* ^2^	0.8725	0.9311

**Table 2:** Parameters of the Kinetic adsorption equation.

Models		DMSNs@MPS@PILs-MIPs	DMSNs@MPS@PILs-NIPs
Pseudo-first-order	*Q*_m_	345.8478	270.4364
	*K*_1_	0.1502	0.1285
	*R* ^2^	0.8016	0.7940
Pseudo-second-orde	*Q*_m_	427.1778	236.9768
	*K*_2_	0.0001	0.0001
	*R* ^2^	0.9949	0.9860
Elovich	A	36.0266	7.9739
	B	0.0098	0.0147
	*R* ^2^	0.9898	0.9852

**Table 3:** Comparison of different methods of imprinting Cyt-C.

Carrier	Functional Monomer	Preparation Strategy	*IF*	*Q*_e_ (mg/g)	Ref.
Cryogel	(MAAP)2-Ce(III)	Bulk Imprinting + Cryopolymerization	4.46	98.33	[46]
SiO_2_	*γ*-CD	Epitope imprinting	3.38	86.47	[29]
SiO_2_	*γ*-CD	Epitope imprinting	3.27	79.56	[47]
Mesoporous silica	Zwitterionic-IL	Epitope imprinting	3.80	249.60	[48]
CN@UIO-66	Zwitterionic-IL	Surface imprinting	6.10	815.00	[49]
Calcium Alginate	Sodium Alginate	electro-spining + molecular imprinting	1.66	3312.00	[50]
DMSNs	PIL	Surface imprinting	2.17	367.86	this work

## Data Availability

The data presented in this study are available on request from the corresponding author. The data are not publicly available due to laboratory policies and confidentiality agreements.

## Supplementary Materials

The following supporting information can be downloaded at: https://www.mdpi.com/article/10.3390/polym18101261/s1, Figure S1: ^1^H NMR spectra of VAIM (a), VCDIM (b), VPIM (c), and VVIM (d); Figure S2: FT-IR spectra of VAIM, VCDIM, VPIM, and VVIM; Figure S3: Predicted binding modes of imidazolium-based ILs with Cyt-C via AutoDock4; Figure S4: Circular dichroism spectra (a1–d1), synchronous fluorescence spectra with Δλ = 15 nm (a2–d2) and Δλ = 60 nm (a3–d3) for the interactions of VAIM, VCDIM, VSIM, and VVIM with Cyt-C; Figure S5: The nitrogen adsorption–desorption isotherms (a) and pore size distribution (b) of DMSNs; Figure S6: The pore size distribution of DMSNs@MPS@PILs-MIPs (a) and DMSNs@MPS@PILs-NIPs (b); Table S1: Equations of adsorption isotherms; Table S2: Equations of adsorption kinetic.
