# Supplementary material for: Harnessing Multi-Anchoring Effects for the Fabrication and Specific Recognition of Surface-Oriented Imprinted Nanospheres for Cytochrome C
Source: Polymers (Basel). 2026 May 21;18(10):1261. doi: 10.3390/polym18101261 (PMC13210750; doi:10.3390/polym18101261)
Supplement: Supplementary file 1 [file polymers-18-01261-s001.zip › polymers-4244372-supplementary.pdf]

Harnessing Multi-Anchoring Effects for the Fabrication and Specific Recognition of Surface-Oriented Imprinted Nanospheres for Cytochrome C

Nan Zhang[1,] *, Yang Qiao[1], Kaishan Yu[1], Jinrong Zhang[1], Pengfei Cui[1], Chengzhao Yang[1], Minglun Li[2,] *

[1] College of Chemistry and Chemical Engineering, Xi'an University of Science and Technology, Xi'an 710054, China; lindajun2022@163.com (Y.Q.); 18709496409@163.com (K.Y.); ZhangJinrong0503@163.com (J.Z.); c1848018709@163.com (P.C.); yyyylolol@163.com (C.Y.)

[2] State Key Laboratory of Polymer Science and Technology, Changchun Institute of Applied Chemistry, Chinese Academy of Sciences, Changchun, 130022, China

* Correspondence: zhangn1123@xust.edu.cn (N.Z.); mlli@ciac.ac.cn (M. L.)

1. Preparation of Ionic Liquid Monomers

1.1 Preparation of VCDIM

β-CD (25.00 g, 22.00 mmol), p-benzenesulfonylimidazole (6.36 g, 28.60 mmol), and 250 mL deionized water were mixed in a conical flask containing a stirring bar. The reaction was allowed to proceed at room temperature (25°C) under vigorous stirring for 4 h. NaOH solution (7.14 g, 32.36 mL) was gradually added to the mixture, and stirring was continued for another 10 minutes. The insoluble solids were filtered out, and the filtrate was collected. The filtrate was neutralized to pH 7 with NH_4Cl (17.29 g, 323.20 mmol) to induce precipitation. The precipitate was collected by filtration, and the solid residue was washed with cold water and acetone. The solid was dried in a vacuum drying oven at 60°C overnight to obtain white solid A. White solid A (6.45 g, 5.07 mmol) was added to a 100 mL round-bottom flask. Then, VIM (1.8 mL, 19.7 mmol) and 15 mL DMF were added to the flask. The flask was transferred to a microwave reactor equipped with a condenser and stirring bar. The mixture was reacted under N_2 atmosphere at 75°C and 80 W for 4 h. After cooling to room temperature, the crude product was precipitated with acetone and dried overnight in a vacuum oven at 50°C to obtain the VCDIM.

1.2 Preparation of VPIM

VIM (1.88 g, 0.02 mol) was dissolved in 10.00 mL of acetonitrile at 50°C. Under vigorous magnetic stirring, 1,3-propane sulfonyl lactone (3.66 g, 0.03 mol) was slowly added to the reactor using a constant pressure dropping funnel. The mixture was stirred at 50°C for 24 h to yield a white precipitate. The precipitate was further purified by washing with a small amount of acetone and lyophilized to obtain the VPSIM.

1.3 Preparation of VVIM

VIM (4.70 g, 50.00 mmol) and VBC (6.50 g, 43.00 mmol) were dissolved in a mixed solution of 20.00 mL water and 20.00 mL ethyl acetate. The reaction was carried out for 24 h. After the reaction, ethyl acetate was used for extraction, and the extract was freeze-dried to obtain the VVPIM.

2. Structural Characterization of VAIM, VCDIM, VPIM, and VVIM

The ^{1}H NMR spectrum and FT-IR spectrum of VAIM, VCDIM, VPIM, and VVIM are shown in **Figures S1 and S2**, respectively.

For VAIM, The ^{1}H NMR (300 MHz, D_2O) spectrum is shown in **Figure S1a**: δ 9.58 (s, 1H), 8.27 (s, 0H), 8.10 (s, 1H), 7.89 (s, 0H), 7.62 (s, 1H), 7.41 (dd, J=15.6, 8.8 Hz, 1H), 6.01 (d, J=15.6 Hz, 1H), 5.44 (d, J=11.2 Hz, 1H), and 5.04 (s, 1H). The FT-IR characterization is shown in **Figure S2**. Characteristic peaks at 3100 cm^{-1}, 1571 cm^{-1}, and 1649 cm^{-1} are assigned to the unsaturated C-H stretching vibration on the imidazole ring, the C=N stretching vibration, and the C=C stretching vibration of the vinyl group attached to the imidazole ring, respectively. In addition, the absorption peaks at 1670 cm^{-1}, 1404 cm^{-1}, and 3116 cm^{-1} correspond to the C=O stretching vibration (amide I band), C-N stretching vibration, and N-H stretching vibration of the amide group, respectively, confirming the successful synthesis of VAIM.

For VCDIM, The ^{1}H NMR (400 MHz, DMSO-d_6) spectrum is shown in **Figure S1b**: δ 8.15 (s, 1H), 7.98 (s, 4H), 7.62 (s, 4H), 7.18 (dd, J=15.8, 8.9 Hz, 5H), 7.02 (s, 4H), 5.50 (d, J=15.8 Hz, 6H), 4.89 (dd, J=8.9, 1.4 Hz, 6H), 4.63 (s, 2H), 4.03 (s, 5H), 3.53–3.46 (m, 16H), 1.89 (s, 3H), and 1.80 (s, 2H). The FT-IR characterization is shown in **Figure S2**. Characteristic peaks at 3100 cm^{-1}, 1571 cm^{-1}, and 1649 cm^{-1} are also attributed to the imidazole ring vibrations. The broad absorption band at 3341 cm^{-1} is assigned to the -OH stretching vibration, while the peak at 1027 cm^{-1} corresponds to the C-O-C stretching vibration. Furthermore, the strong absorption band at 1730 cm^{-1} is ascribed to the C=O stretching vibration of the ester group, indicating the successful formation of VCDIM.

For VPIM, The ^{1}H NMR (400 MHz, DMSO-d_6) spectrum is shown in **Figure S1c** (400 MHz, DMSO-d_6): δ 9.47 (s, 1H), 8.18 (s, 1H), 7.94 (s, 1H), 7.29 (dd, J=15.7, 8.8 Hz, 1H), 5.95 (dd, J=15.6, 2.2 Hz, 1H), 5.41 (dd, J=8.7, 2.2 Hz, 1H), 4.34 (t, J=7.0 Hz, 2H), 2.44 (t, J=7.1 Hz, 2H), and 2.13 (p, J=7.0 Hz, 2H). The FT-IR characterization is shown in **Figure S2**. In addition to the characteristic imidazole ring absorptions at 3100 cm^{-1}, 1571 cm^{-1}, and 1649 cm^{-1}, the absorption bands at 1188 cm^{-1} and 1048 cm^{-1} are assigned to the asymmetric and symmetric stretching vibrations of the -SO_3^- group, respectively, confirming the successful synthesis of VPIM.

For VVIM, The ^{1}H NMR (400 MHz, DMSO-d_6) spectrum is shown in **Figure S1d** (300 MHz, DMSO-d_6): δ 9.68 (s, 1H), 8.25 (d, J = 5.0 Hz, 1H), 7.96 (s, 1H), 7.56–7.43

(m, 4H), 7.38–7.28 (m, 2H), 6.75 (dd, J = 13.2, 8.2 Hz, 1H), 5.94 (dd, J = 29.1, 12.4 Hz, 2H), 5.46 (s, 2H), and 5.31 (d, J = 8.1 Hz, 1H). The FT-IR characterization is shown in **Figure S2**. The characteristic imidazole ring absorptions appear at 3100 cm^{-1}, 1571 cm^{-1}, and 1649 cm^{-1}. The absorption band at 825 cm^{-1} is attributed to the out-of-plane bending vibration of C-H on the meta-disubstituted benzene ring, further verifying the successful synthesis of VVIM.

3. **Quantum Mechanical Calculation Method for the Interaction Mechanism Between Ionic Liquids and Cyt-C**

3.1 Construction and Optimization of Monomer Structures via GaussView

The initial geometric configurations of the template molecule and four imidazolium-based ionic liquid monomers were constructed using GaussView 05. Herein, a representative polypeptide chain fragment of Cyt-C (with the characteristic amino acid sequence A-Y-L-K-V-L-N-D-S) was selected as the template molecule instead of the complete protein structure. This is because the computational cost of the DFT method increases exponentially with the number of atoms when dealing with macromolecular systems, making it impossible to complete high-precision optimization of the complete protein structure within a reasonable computational time. Therefore, a polypeptide chain fragment that can characterize the distribution characteristics of key recognition sites and functional groups was chosen as the simplified model. Subsequently, the geometric structures of the Cyt-C polypeptide chain and the four ionic liquid monomers were optimized separately using the Gaussian 16 program. The density functional theory (DFT) method in quantum mechanical calculations was adopted with the B3LYP/6-31G basis set, and the DFT-D3 dispersion correction was introduced to improve the description accuracy of weak interactions.

3.2 Prediction of Monomer–Protein Interaction Modes via AutoDock

The stable configurations of the Cyt-C polypeptide chain (optimized by Gaussian 16) combined with each ionic liquid monomer were selected for molecular docking simulations using AutoDock software. Each ionic liquid monomer-Cyt-C polypeptide complex system was run independently 10 times, with all other parameters set to their default values. After docking, the configuration with the lowest binding energy (maximum absolute value) was regarded as the optimal binding configuration between the ionic liquid monomer and the Cyt-C polypeptide, based on which the interaction modes between ionic liquids and the Cyt-C polypeptide were predicted.

4. Effects of Small-Molecule Ionic Liquids on the Native Conformation of Cyt-C

To further clarify the influence of small-molecule monomers on the conformational stability of native proteins, the interactions between four small-molecule ionic liquid monomers (VAIM, VCDIM, VSIM, and VVIM) and Cyt-C were systematically investigated. Circular dichroism (CD) spectroscopy and synchronous fluorescence spectroscopy were employed to analyze the changes in the secondary and tertiary structures of Cyt-C, and the relevant results are presented in **Figure S4**.

The CD spectroscopic results (**Figure S4(a1–d1)**) show that native Cyt-C exhibits characteristic absorption peaks of the α-helix structure at 208 nm and 222 nm. When the mass ratio of monomer to Cyt-C was less than 1:1, the CD peak profiles of each system changed slightly, indicating that the native conformation of the protein could be well maintained at low monomer concentrations. However, when the mass ratio exceeded 1:1, varying degrees of changes in peak profiles were observed for all four small-molecule monomer systems, suggesting that the α-helix structure of the protein was significantly perturbed. This phenomenon may be attributed to the fact that small-molecule monomers tend to penetrate the interior of the protein and thereby destroy its structure.

Synchronous fluorescence spectroscopic results further revealed the effect of small-molecule monomers on the microenvironment of the tertiary structure of Cyt-C (**S4(a2–d2) and S4(a3–d3)**). With the increase of monomer dosage, the fluorescence intensity of VAIM and VSIM systems gradually increased. This may be ascribed to the formation of hydrogen bonds or electrostatic interactions between the two monomers and polar amino acid residues on the protein surface, which enhanced the microenvironmental rigidity around partial tyrosine and tryptophan residues. In contrast, with the increasing monomer dosage, the VCDIM and VVIM systems displayed more obvious red-shift of emission peaks and distinct changes in peak shape. Generally, the fluorescence red-shift indicates that the aromatic amino acid residues inside the protein are gradually exposed to the aqueous environment, implying the loosening of the internal hydrophobic region of the protein. Among them, VCDIM could enhance the perturbation to the internal structure of the protein via the inclusion interaction between the β-cyclodextrin cavity and the hydrophobic region of the protein. Benefiting from its highly hydrophobic vinylphenylethyl structure, VVIM tends to insert into the hydrophobic core region of the protein, resulting in the exposure of intrinsic chromophores and the alteration of the internal microenvironment. Consequently, both monomers exert a strong perturbation effect on the tertiary structure of the protein. In summary, small-molecule ionic liquid monomers are prone to induce structural destruction of proteins, which probably originates from the excessively strong local

interaction at a single action site and the easy access of small-molecule monomers to the internal hydrophobic region of proteins.

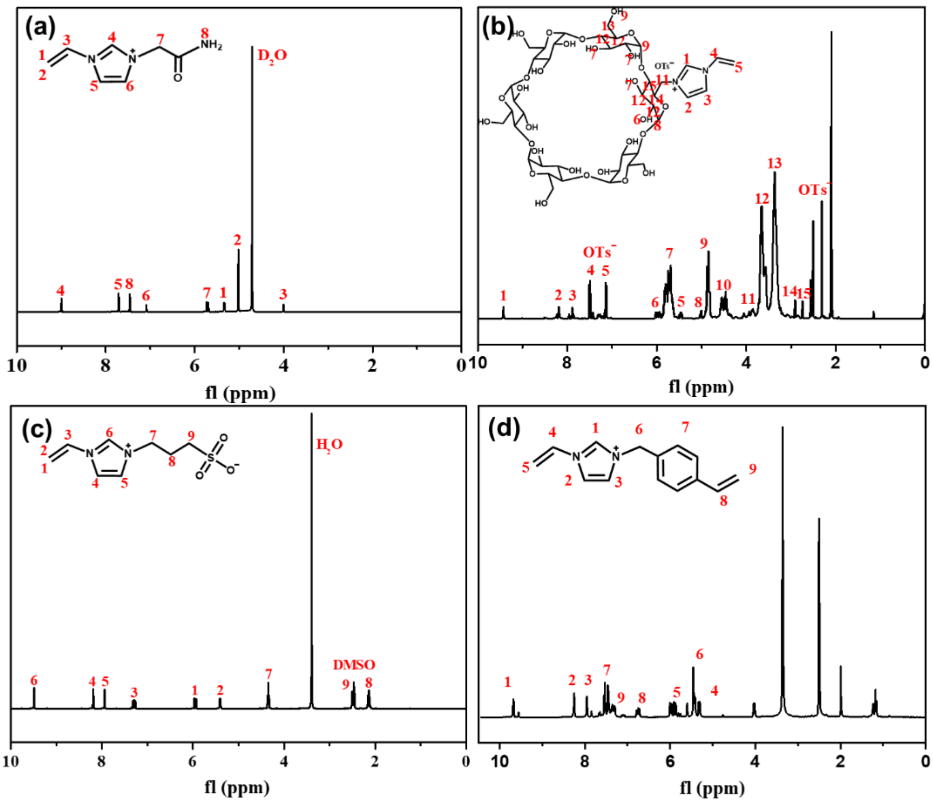

Figure S1. ^{1}H NMR spectra of VAIM (a), VCDIM (b), VPIM (c), and VVIM (d).

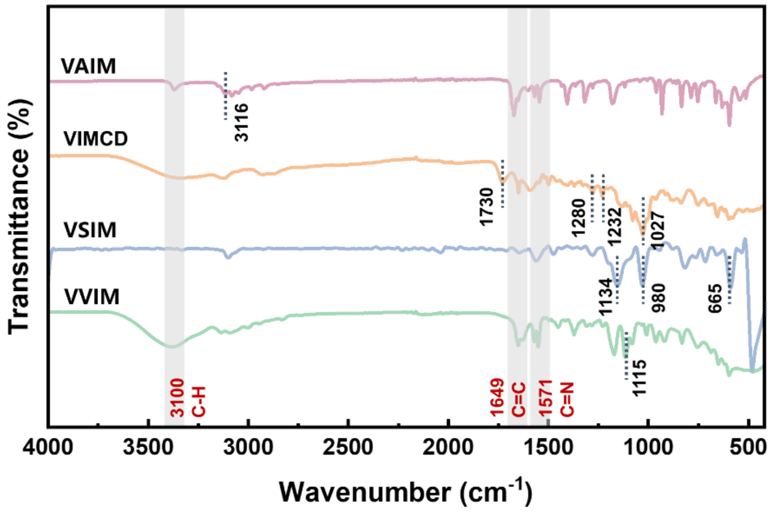

Figure S2. FT-IR spectra of VAIM, VCDIM, VPIM, and VVIM.

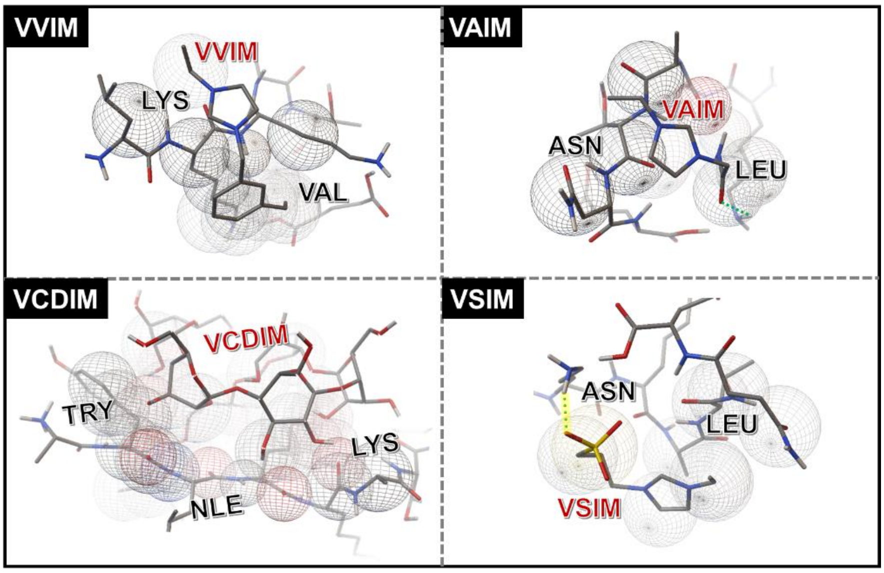

Figure S3. Predicted binding modes of imidazolium-based ILs with Cyt-C via AutoDock.

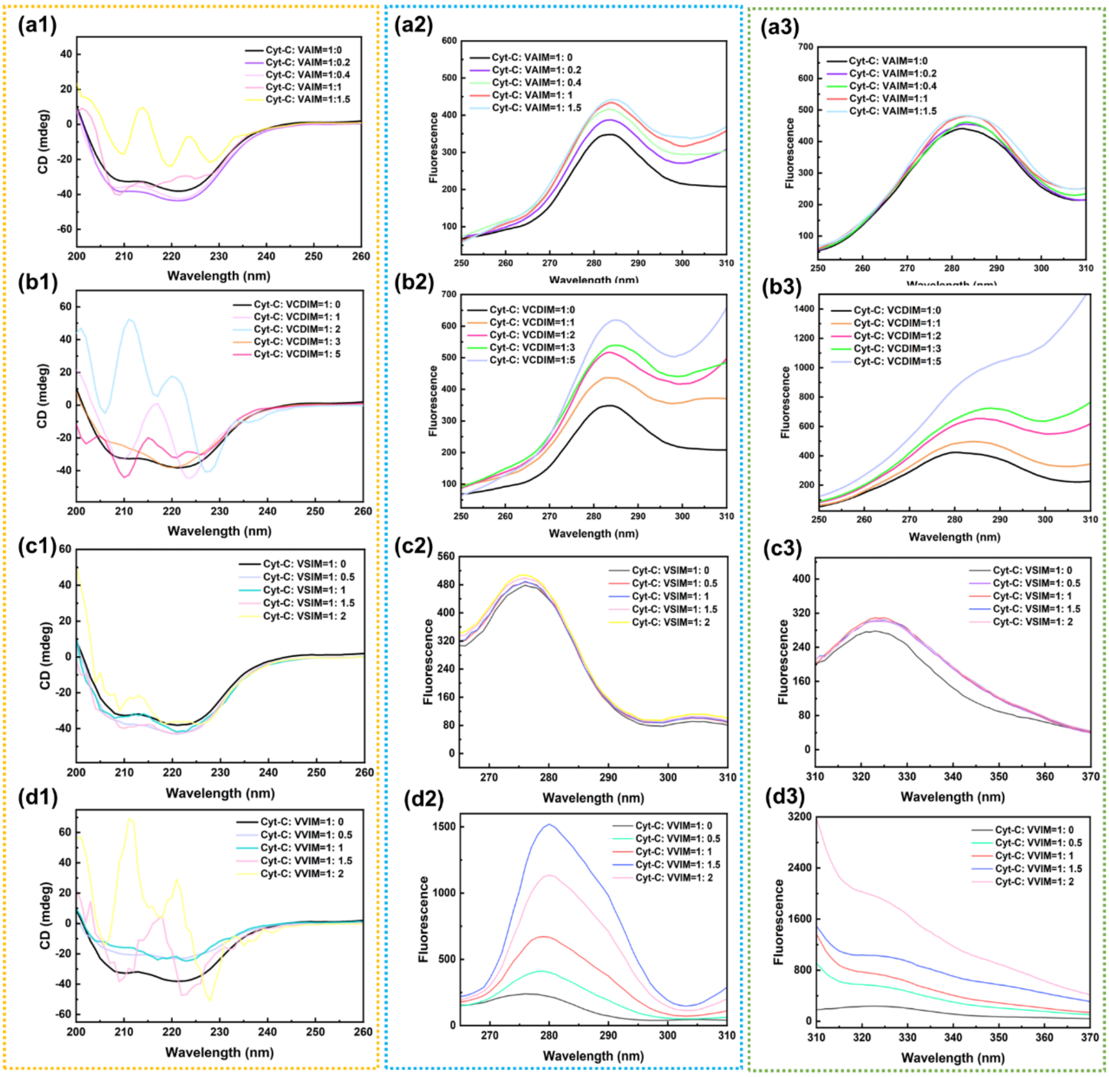

Figure S4. Circular dichroism spectra (a1–d1), synchronous fluorescence spectra with Δλ = 15 nm (a2–d2), and Δλ = 60 nm (a3–d3) for the interactions of VAIM, VCDIM, VSIM, and VVIM with Cyt-C.

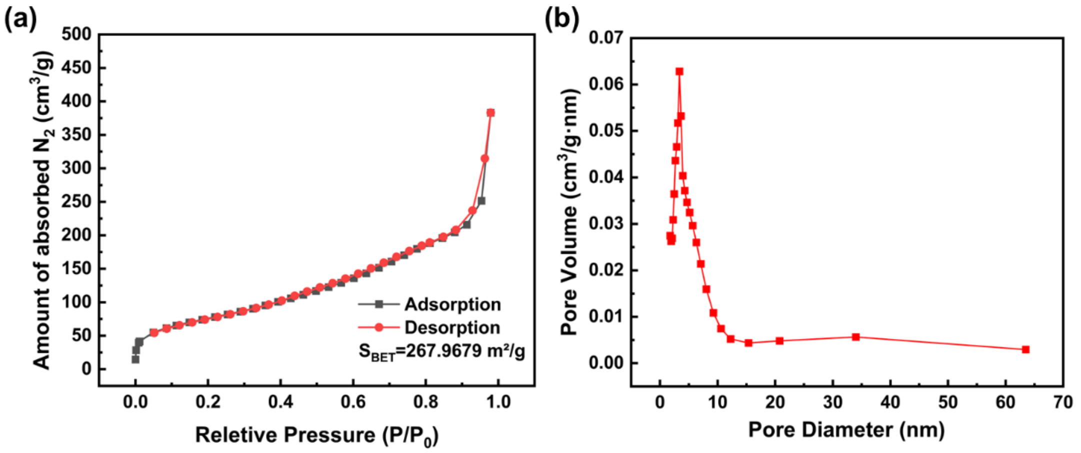

Figure S5. The nitrogen adsorption–desorption isotherms (a) and pore size distribution (b) of the DMSNs.

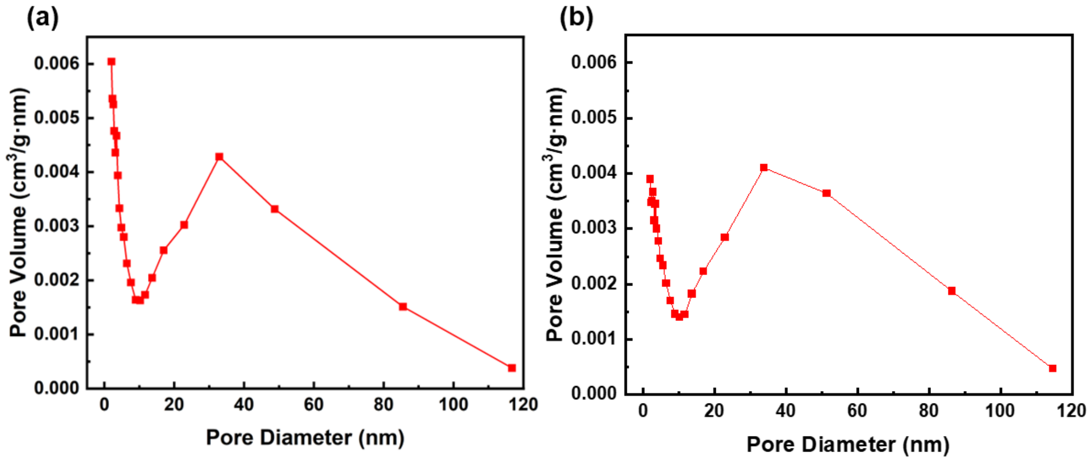

Figure S6. The pore size distribution of DMSNs@MPS@PILs-MIPs (a) and DMSNs@MPS@PILs-NIPs (b).

Table S1. Equations of adsorption isotherms

Isotherm Models	Equation	Parameters
Langmuir	$\frac{C_e}{Q_e} = \frac{1}{Q_m}C_e + \frac{1}{k_L Q_m}$	Q_e: equilibrium adsorption capacity, mg/g; C_e: equilibrium concentration, mg/mL; Q_m: maximum adsorption capacity, mg/g; K_d: Langmuir dissociation constant of adsorption, mol/L;
Freundlich	$\ln Q_e = \frac{1}{n} lnC_e + \ln K_F$	K_F: Freundlich adsorption equilibrium constant, mL/mg; n: constant related to adsorption intensity;
Temkin	$Q_e = BlnA + BlnC_e$	A (mL/mg) and B: the Temkin equilibrium constants for adsorption.

Table S2. Equations of adsorption kinetics

Kinetic Models	Equation	Parameters
PFO	$ln(Q_e - Q_t) = ln\,Q_e - \mathrm{k}_1 t$	Q_e: equilibrium adsorption capacity, mg/g; Q_t: adsorption capacity at time t, mg/g; k_1: adsorption rate constant of the PFO kinetic equation, 1/min;
PSO	$\frac{t}{Q_t} = \frac{1}{\mathrm{k}_2 Q_e^2} + \frac{t}{Q_e}$	k_2: adsorption rate constant of the PSO kinetic equation, 1/min;
Elovich	$Q_t = \ln(A * B * t + 1)\,/B$	A and B are rate constants for the Elovich model.
